# Bilateral emphysematous pyelonephritis and emphysematous cystitis: Potential catastrophic complication of mid-urethral sling surgery

**DOI:** 10.1016/j.eucr.2025.103263

**Published:** 2025-11-01

**Authors:** Ethan Layne, Joseph Ford, Shilo Rosenberg

**Affiliations:** a USC Institute of Urology and Catherine and Joseph Aresty Department of Urology, Keck School of Medicine, University of Southern California, Los Angeles, CA, USA; b Kaweah Delta Hospital, Visalia, CA, USA

**Keywords:** Urinary retention, Mid-urethral sling, Emphysematous pyelonephritis, Emphysematous cystitis, Surgical complications

## Abstract

To our knowledge we present the first case of bilateral emphysematous pyelonephritis (EPN) and emphysematous cystitis (EC) diagnosed, in a diabetic patient with urinary retention, five days after ambulatory robotic hysterectomy and mid-urethral sling (MUS). Her urethral catheter was removed prior to discharge. This report describes a potentially catastrophic event that should have been prevented. Adhering to appropriate voiding trial protocols should continue to be an essential part of the discharge process.

## Highlights

• Monitor post-op voiding event after surgery with bladder outlet obstruction risk.
• Optimal approach to patients with emphysematous pyelonephritis is not clear.
• Classifications systems should guide but not decide treatment.
• Long term renal function may normalize after bilateral emphysematous pyelonephritis.

## 1 Introduction

Emphysematous tissue infections are necrotizing severe and potentially life-threatening conditions. In the urinary tract these infections are often associated with female and diabetics. The combination of poor tissue perfusion with high glucose levels, obstruction, immunosuppression and gas producing pathogens are thought to be the ingredients necessary for the propagation of this event. Females are thought to affected more than men due to their increased susceptibility for urinary tract infections (UTI).

Historically, intravenous antibiotics and emergency nephrectomies were the standard of care with a reported mortality rate of 40–50 %. However, over the last three decades, due to better critical care, improved imaging, increased availability and improvement in interventional radiology, mortality has significantly decreased with better recovery of renal function. More recent studies report mortality rates of 11.5–13.5 %, when treated by medical management and percutaneous nephrostomy drainage.^1^^,^^2^

Post-operative urinary retention (POUR) occurs when patients are unable to void after surgery and may be as high as 20 %.^3^ The cause of this condition is usually related to the type of anesthesia and its effect on the detrusor muscle and, if present, the extent of pelvic nerve injury. The post-operative patient often lacks intact sensorium and therefore diagnosing POUR can be a challenge. The incidence of urinary retention after MUS surgery has been reported from 2 to 19 %.^4^ Urinary retention in this subset of patients is mainly due to obstruction from the sling.

With increased use of minimally invasive approach for pelvic surgeries and implementation of enhanced recovery protocols, same day discharges are becoming common.^3^ To make same day discharges advantageous for the patient and healthcare systems, and safe, adherence to strict “voiding protocols” are of profound importance.

We present a case of concurrent bilateral EPN and EC diagnosed, in a diabetic female patient, in urinary retention, five days after robotic hysterectomy and MUS implantation.

## 2 Case report

**Fig. 1:**
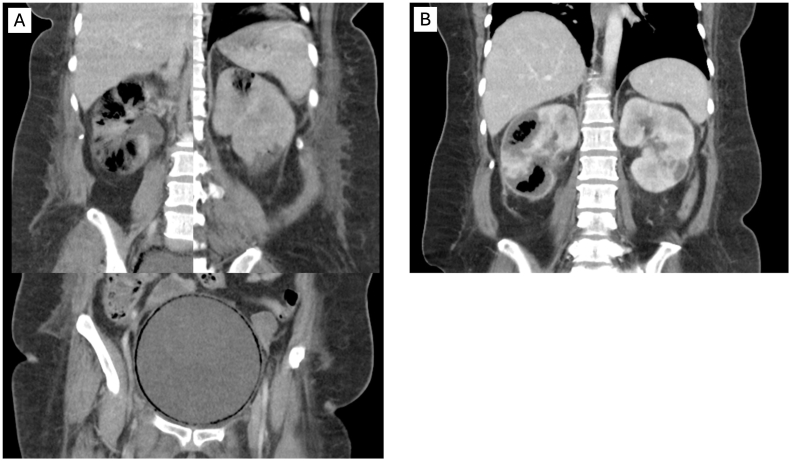
Imaging; CT abdomen pelvis. 1a)Bilateral Imaging CT abdomen pelvis on admission. 1b) CT abdomen and pelvis carried out 3 weeks after admission, after right percutaneous nephrostomy tube was accidently dislodged.

A 54-year-old, diabetic, female patient presented to the emergency room (ER) for altered mental status, lethargy and hyperglycemia five days after ambulatory robotic hysterectomy and MUS implantation at another hospital. She was discharged without a urethral catheter. She stated that since discharge, she was straining to void, had dribbling of urine and had urinary frequency. Three days prior to her ER admission she developed lower abdominal distension and pain. In the ER patient was afebrile with tachycardia 116 beats/minute and blood pressure 90/57. Laboratory results demonstrated acute renal failure, lactic acidosis, ketoacidosis, and thrombocytopenia of 7 x10ˆ3/mcL (Table 1). Computer tomography of the head, chest, abdomen and pelvis was significant for bilateral EPN and EC and urinary retention (Fig. 1a). Intravenous fluids and antibiotics were initiated. An 18F urethral catheter was placed and drained 1250 mL of “tea colored” urine. Urology consult recommended bilateral renal drainage by percutaneous nephrostomies following platelets replacement therapy (PRT). Once PRT was initiated, a patient developed septic shock. She was intubated and vasopressors started. Given her hemodynamic instability she was admitted to the ICU for optimization. A day later, once thrombocytopenia improved, interventional radiology inserted a single 8F right PCN. This was 26 hours from her initial presentation to the ER. She spent eight days in the ICU, seven on vasopressors. On the 22nd day of admission her right PCN was accidently dislodged. Since she was doing well clinically no further interventions were required. However, a CT scan of the abdomen and pelvis that was ordered still demonstrated gas within the right renal parenchyma (Fig. 1b). The patient was hospitalized a total of 29 days, and she failed a voiding trial before discharge. She subsequently underwent sling incision and resumed normal voiding 3 months after initial MUS implantation. Three years following her admission she has normal renal function with creatinine 0.83 mg/dl (GFR 83) with minimal LUTS, no SUI and normal bladder emptying.

**Table 1:** Laboratory values upon admission.

Parameter	Value
White Blood Cells	11.37 mcL
Hemoglobin	6.8 gr%
Platelets	7 × 10^3^ mcL
Creatinine	5.09 mg/dL
Lactic Acid	4.8 mmol/L
Sodium	122 mmol/L

## 3 Discussion

EPN is a necrotizing life threating infection. Simultaneous bilateral EPN and EC are a rare but documented occurrence. Factors associated with the propagation of these necrotizing infections are high glucose tissue levels, obstructive uropathy, immunosuppression and hypertension. Our patient's HBG A1c prior to surgery was 7.2 %. We hypothesize that in our case infra-vesical obstruction, due to MUS surgery, led to increased pelvicalyceal pressure thereby reducing renal parenchymal perfusion in an already ischemic environment. The Huang-Tseng clinico-classification system is the most prevalent for EPN (Table 2). It uses CT imaging to classify EPN into 4 classes. This classification system, in concert with risk factors such as altered mental status, septic shock, thrombocytopenia and acute renal failure, has been shown to correlate with prognosis and guide treatment planning. However, these classification systems should only guide and not decide clinical approach since our patient recovered well with only a single right PCN inserted but clinically she would have been considered a class 4 EPN with all risk factors present that would have mandated bilateral PCN insertion. Perhaps it's not the number of renal units that should dictate the severity of EPN, but the percentage of total renal parenchyma involved in the infectious process. Another question that we struggled with was when to remove the PCN. Her right PCN was spontaneously (thankfully) dislodged three weeks into her admission and the CT scan that was subsequently obtained still demonstrated gas within the right renal parenchyma. We believe that a PCN may be removed once the overall clinical condition of the patient improves. The mere presence of gas within the renal parenchyma alone appears to be a suboptimal indication for intervention. Our patient has normal renal function 3 years status post bilateral EPN infection. To the best of our knowledge there are no studies addressing long term renal function after EPN. Our case suggests that normal renal function may be expected even after an episode of bilateral EPN.

**Table 2:** Emphysematous Pyelonephritis classification (Huang- Tseng).

Class	Description
Class I	Gas in collecting system only
Class II	Parenchymal gas only
Class IIIa	Extension of gas into perinephric space
Class IIIb	Extension of gas into pararenal space
Class IV	EPN in solitary kidney, or bilateral disease

A voiding trial is not the mere removal of an indwelling catheter. The patient's voiding event should be meticulously monitored immediately post-operatively and often even longer. POUR is a relatively common phenomenon. In diabetics, as in our case, it is even more common. Postoperative recovery unit providers should be educated on possible complications related to patients undergoing ambulatory pelvic surgeries especially with a potential for bladder outflow obstruction. The author's experience is that nurses carrying out voiding trials, in the postoperative units, often emphasize urinary output and not postvoid residuals which together with patient's voiding event are the most important factors to monitor before a safe discharge. Ultimately it is the responsibility of the surgeon to ensure a safe discharge.

## 4 Conclusion

To our knowledge this is the first case of bilateral EPN and EC developing as a result of missed urinary retention in a patient after robotic simple hysterectomy and implantation of a MUS. Voiding trial should be meticulously followed according to enhanced recovery protocols. Clinico-classification systems should guide but not decide treatment approach as they do not completely correlate with clinical outcomes. Long term renal function may normalize even in diabetics with a history of bilateral EPN.

## CRediT authorship contribution statement

**Ethan Layne:** Visualization, Writing – review & editing. **Joseph Ford:** Writing – review & editing. **Shilo Rosenberg:** Supervision, Writing – original draft.

## Funding

None.

## COI

None.
